# CHOPIN: a web resource for the structural and functional proteome of *Mycobacterium tuberculosis*

**DOI:** 10.1093/database/bav026

**Published:** 2015-03-31

**Authors:** Bernardo Ochoa-Montaño, Nishita Mohan, Tom L. Blundell

**Affiliations:** ^1^Department of Biochemistry, University of Cambridge, Sanger Building, 80 Tennis Court Road, Cambridge CB2 1GA, UK and ^2^Department of Biotechnology, Indian Institute of Technology Madras, Chennai, Tamil Nadu 600036, India

## Abstract

Tuberculosis kills more than a million people annually and presents increasingly high levels of resistance against current first line drugs. Structural information about *Mycobacterium tuberculosis* (Mtb) proteins is a valuable asset for the development of novel drugs and for understanding the biology of the bacterium; however, only about 10% of the ∼4000 proteins have had their structures determined experimentally. The CHOPIN database assigns structural domains and generates homology models for 2911 sequences, corresponding to ∼73% of the proteome. A sophisticated pipeline allows multiple models to be created using conformational states characteristic of different oligomeric states and ligand binding, such that the models reflect various functional states of the proteins. Additionally, CHOPIN includes structural analyses of mutations potentially associated with drug resistance. Results are made available at the web interface, which also serves as an automatically updated repository of all published Mtb experimental structures. Its RESTful interface allows direct and flexible access to structures and metadata via intuitive URLs, enabling easy programmatic use of the models.

**Database URL**: http://structure.bioc.cam.ac.uk/chopin

## Introduction

Recent progress in the global fight against tuberculosis has been modest and the burden of the disease is still great, with over one million deaths and more than eight million new infections annually (http://www.who.int/tb/publications/global_report/en/). One of the major challenges ahead is tackling the rise of multi-drug resistant strains of the bacterium, which requires the development of new and more effective drugs and a better identification and understanding of potential molecular targets.

The actions of drugs are determined by their chemical interactions with macromolecules, particularly proteins, and thus the detailed information about them provided by structural insights is especially valuable for their design. However, experimental determination of protein structures is often laborious, expensive and a difficult undertaking, such that only about 10% of the 4000 protein sequences that constitute the *Mycobacterium tuberculosis* (Mtb) proteome (1, 2) have been structurally determined (3). Fortunately, recent progress in bioinformatics methods and computing power, as well as the dramatic growth of biological data in general repositories, has made the prediction of structures an increasingly viable alternative for the provision of structural information on a genomic scale, which can often be useful despite the limitations in accuracy and reliability of homology modelling relative to experimental determination.

There have been various efforts to generate wholesale models for entire organism proteomes, such as MODBASE (4) and Genome3D (5), including one for Mtb (6). Their focus, however, has been to maximize the genome’s coverage using a single ‘best’ template, as this is generally sufficient to derive general information about fold and function, as Anand et al. (7) have done using MODBASE. What constitutes ‘best’, however, is difficult to determine objectively, since there are multiple factors that affect a template’s suitability, such as similarity to the target, coverage and experimental quality, which require a subjective balancing decision. The use of multiple templates in modelling helps to take advantage of the information present in all of them, but this must be done with care. Template libraries may include the entirety of the Protein Data Bank (PDB; 8), or a filtered non-redundant subset of it, or structures in a processed form, such as individual domains according to classifications like SCOP (9) or CATH (10). However, often overlooked is the matter of the biological context of the templates, such as whether they are in complexed form with other subunits or bound with cofactors, drugs or other molecules. This is of relevance since much of the redundancy of sequences in the PDB is due to the study of multiple forms of proteins, which manifest in conformational differences according to context. While some of these might be too subtle to exert meaningful influence on a homology model, others can be quite drastic, so it is important to take it into consideration, especially when using multiple templates.

As mentioned above, the development of drug resistance is one of the main reasons for the need for novel drugs against Mtb, and thus understanding and predicting the functional effect of polymorphisms on their targets is of prime importance in their development. As with modelling, computational methods are increasingly able to step up and provide insight where more expensive experimental methods are unable to, with programs such as SIFT (11), Site Directed Mutator (SDM; 12, 13) and PolyPhen (14) and mutant Cutoff Scanning Matrix (mCSM; 15) being developed in recent years.

In this work, we present CHOPIN, a database built on an automated, high-throughput modelling pipeline using multiple templates, annotated according to functional state. CHOPIN also incorporates an analysis using the computer programs SDM and mCSM of polymorphisms that are possibly related to drug resistance. All the information, together with an up-to-date compendium of Mtb structures, is made easily accessible from a web interface.

## Methods

**Figure 1.**
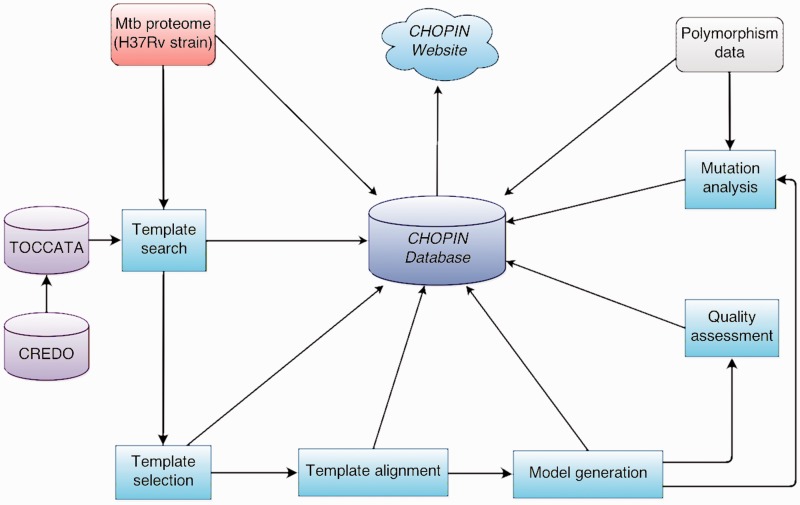
Overview of the CHOPIN modelling pipeline.

An overview of the modelling pipeline is illustrated on Fig. 1. It begins with the set of FASTA-formatted sequences from the H37Rv reference genome, as obtained from the Tuberculosis Database (TBDB) website (16) and outputs a set of models and alignments, together with a relational database of the data necessary for the web interface.

The pipeline is based on the Ruffus pipelining module (17) for Python, which allows most processes to be automated and parallelized, making the method generally applicable to the high-throughput modelling of proteomes or other large sets of sequences.

### TOCCATA

TOCCATA (Ochoa-Montaño B, Bickerton R and Blundell TL, manuscript in preparation, http://structure.bioc.cam.ac.uk/toccata) is a database of templates developed in conjunction with CHOPIN, which underlies the template identification and selection process in the pipeline. The database incorporates all domains from SCOP 1.75A and CATH 3.5, forming a consensus ‘profile’ whenever the domains of a SCOP family can be reasonably matched to a CATH superfamily, otherwise keeping them in their respective [super]families. It was decided to pair CATH superfamilies with SCOP families instead of superfamilies as this leads to more consistent joint profiles of a manageable size.

Full chains consisting of multiple domains are also grouped into profiles according to their domain composition, thus enabling the modelling of multi-domain targets with a plausible spatial relationship between the domains by adopting that of a homologue. All PDB files assigned to a profile are clustered using CD-HIT (18) according to sequence similarity at different thresholds: 50, 70, 90 and 95%. A representative from each sequence cluster at a certain threshold is selected from each TOCCATA profile to generate a FUGUE profile file, which is used by the fold recognition program FUGUE (19) for homology searches. The identity threshold is variably adjusted to keep the number of sequences included at 25 or less, whenever possible. This subset of sequences is aligned using BATON, an in-house, streamlined version of COMPARER (20) and, in the case of profiles with <25 sequences, it is further enriched with sequence homologues using PSI-BLAST (21).

Each chain and domain in TOCCATA is annotated using the CREDO database (22) with information about its binding status to biologically relevant ligands and other chains, and its experimental quality. TOCCATA assigns a ‘quality score’ (Qscore) based on the combination of various measures according to the following equation:

$$\left(\frac{1}{\text{Resolution}}+\left(0.1-\text{Rfactor}\right)\right)\times \left(1-\text{missing residue fraction}\right)$$

It is similar to the AEROSPACI score used by the ASTRAL compendium (23), which is also stored, but Qscore eschews stereochemical checks in favour of considering missing regions of the structure, which should be minimized for modelling purposes.

### Template identification

The identification of templates for each target achieved using the program FUGUE (19) on the TOCCATA database of profiles. Target sequences are pre-processed into a query alignment by searching for homologues using PSI-BLAST on the UniRef50 (24) database and subsequently aligned with MAFFT (25). In the case of sequences of length over 300, which are likely to contain more than one domain, a pre-search step is also performed using HMMER (26) on the PFAM database (27) to determine probable domain boundaries, if any. Any resulting sub-sequences are searched individually together with the full sequences against the set of single-domain profiles. Whenever there are significant FUGUE hits (i.e. profiles with *Z*-scores of at least 4.0) for different sub-sequences or regions of a sequence, TOCCATA is queried for multi-domain profiles that include the relevant combination of domains and the full sequence is then compared against any available results. Matches that span multiple domains in this way can then be used to build models that combine them in a spatially plausible way.

Given TOCCATA’s conservative grouping of SCOP and CATH hierarchies and the inherent similarity between several families, there are often multiple significant hits corresponding to various closely related profiles. To avoid the increased redundancy, complexity and resource requirements, only the hit with the highest *Z*-score for every matched region of the sequence was selected for further processing; however, exceptions are made if a somewhat lower scoring hit has at least 25% larger coverage than the better one, since they may include additional domains or significant secondary structure elements.

For every selected hit, the target sequence is cut to the matched range for the following steps in the pipeline.

### Template selection

Once a TOCCATA profile is selected for a sequence or part of it, the first step is to determine the similarity of the target sequence to the representatives of clusters at 95% sequence identity. To achieve this, the percentage identity (PID) to each representative sequence is calculated, after aligning it to the target using FUGUE. All sequences from the clusters that have a PID more than 20% below the maximum one are discarded, unless they happen to have the greatest coverage to the target sequence and have a PID of at least 50%.

Templates from the remaining clusters are then classified in different groups according to their TOCCATA annotation of ligand binding and oligomerization state. Upto five different groups are populated with the available templates: liganded-monomeric, liganded-complexed, apo-monomeric, apo-complexed and any, which includes templates regardless of their status. For any group that has more than five templates after this, a pruning procedure is applied to reduce the number to at most five, by iteratively removing the template with the lowest similarity to the target from the pair with the highest similarity between themselves. This has the effect of removing the most redundant templates and preserving the structural diversity.

### Template alignment

In the cases where more than one template is available, they are aligned using BATON. An independent superposition is then performed on the alignment thus generated using the program THESEUS (28). This was done to obtain the transformation matrix for later use, in addition to the program’s own stated advantages, which include a maximum likelihood optimisation procedure that ensures that the variable regions of a structure have a reduced weight in the superposition.

In the case of the alignments for liganded groups, the templates are further post-processed to include biologically relevant ligands in their binding sites, with the goal of enabling their modelling into the target. However, with multiple templates, it is sometimes the case that they will have incompatible ligands that cannot all be modelled at the same time, such that a selection procedure becomes necessary. Since the purpose of a general model is to depict it in a natural or typical state, the default procedure is to select the most frequent ligands under the assumption that they are a common component of the protein family. Given that a protein may bind more than one copy of a given molecule at different sites, they need to be discriminated by clustering them by their geometric centroids in the superposed structures. Once this is done, the alignment is modified so that the model will inherit any ligands present in at least half of the templates.

### Modelling and quality assessment (QA)

MODELLER 9v10 is used on all generated alignments to produce three models with fast refinement and NDOPE (29) and GA341 (30) assessment methods enabled. In addition to these built-in methods, the models are also subjected to processing by MolProbity (31) and an in-house secondary structure agreement (SSAG) assessment based on the work by Eramian et al. (32)

While MolProbity is designed to validate structures generated by experimental methods and is not meant to establish the accuracy of a theoretical model, it was considered that the stereochemical evaluation it provides is nevertheless useful and complementary to the other methods. In particular, the MolProbity score is designed to provide an approximation of crystallographic resolution at which the various parameters would be found, which can be valuable provided the other estimates are good.

The SSAG assessment is based on the agreement between the assigned secondary structure of the model with that predicted from the sequence by PSIPRED, a relationship that has been observed to correlate strongly with the correctness of the model. SSAG is used in two varieties, where the scores are referred to as PSIPRED_PERCENT_ and PSIPRED_WEIGHT_:

$${\text{SSAG}}_{\text{frac}}=\frac{{\text{PredSS}}_{\text{inc}}}{N_{\text{res}}}{\text{SSAG}}_{\text{weight}}=\frac{\sum_{i=1}^{R_{\text{inc}}}{\left(C_{i}\right)}^{2}}{N_{\text{res}}}$$

where PredSS_INC_ is the number of incorrectly predicted residues; *N*_res_ is the total number of residues in the sequence; *C_i_* is the confidence value (0–9) of the prediction for residue *i*, with *R*_inc_ being all residues with mismatched predictions.

The different scores are combined into a general guide of the estimated quality rating that ranges from 0–1 (Poor) to 4 (Great). Models are assigned an initial score of 2 and either gain or lose points according to their satisfaction of thresholds of their various scores. For NDOPE, the score is increased by one point if its value is −0.5 or lower and decreased if the value is 0.5 or higher, while for GA341, a point is lost for a value below 0.6 and gained for one of 0.98 or higher. In the case of MolProbity, a score below 3.0 will increase the score and one higher than 4.2 will decrease it. Since both varieties of the SSAG score are correlated, a single score adjustment is done when either of their thresholds is crossed. For SSAG_FRAC_, a point is subtracted if the score is ≥0.5 or added if it is <0.2, whereas for SSAG_WEIGHT_ the thresholds are ≥20 and <10, respectively. For these scores, the thresholds were determined by analysing a set of 4725 non-redundant high quality structures [selected from the PISCES (33) culled PDB list with a resolution <2.5 Å] and setting the value for the subtraction of points at approximately the 99th percentile (after rounding) and for the addition at either at two standard deviations (SSAG_WEIGHT_) or the average (SSAG_FRAC_; the difference being due to their distinct distributions).

Additionally, to rank models a more detailed adjustment is performed on the combined score by assigning it fractional bonus points according to a set of more fine-grained thresholds. The set of thresholds used is presented in Supplementary Table S1.

Due to this threshold-based approach, it should be noted that ‘poor’ models should not be taken as necessarily incorrect as a whole, especially in cases where the FUGUE *Z*-score is high, but rather that they have raised flags on some of the assessment criteria and should be regarded with care. While a poor rating can be indicative of alignment issues, particularly in cases where other states have higher ratings, it may also be the case that the structures include some atypical feature (e.g. intrinsically disordered regions, included ligands, domain swaps or long interacting stretches), such that they fall outside of what the assessment methods are trained to deal with. This can be of relevance for models in the ‘liganded’ category, since the introduction of the modelled ligand can influence the assessment compared with a seemingly equivalent alignment on a different category.

Finally, in addition to the fast, locally run QA methods that constitute the general quality estimate, the website also allows for the on-demand submission of any model to the QMEAN server (34), a well-established, top-performing method at recent Critical Assessment of Structure Prediction exercises (35).

### Mutation analysis

#### Datasets

Sequences for polymorphisms were obtained from two sources. The Broad Institute has sequenced the genomes of several strains of Mtb, including three from Kwazulu-Natal (KZN, a region in South Africa), which display a mix of drug sensitivity (DS), multiple drug resistance (MDR) and extensive drug resistance (XDR) and have recently been subject to a genomic analysis (36). A total of 471 polymorphisms between these strains and the reference one (F11) has been made available on their website (http://www.broadinstitute.org/annotation/genome/mycobacterium_tuberculosis_spp/ToolsIndex.html). The TB Drug Resistance Mutation Database (TBDReaMDB; 37) compiles mutations related to drug resistance determined by validated experimental methods published in the literature and provide both a complete list as well as a ‘high confidence’ subset. Non-synonymous polymorphisms leading to residue changes on the KZN strains and the TBDReaMDB high confidence set were used for further processing, providing a total of 263 possible mutations.

For the source structural data, experimentally solved structures were preferentially used when available, otherwise any models spanning the mutated residue were utilized. Of all mutations, 147 were found to lie within the range of available models, of which 36 were in experimental structures and 111 in homology models.

Since the mutations of the Broad Institute set are expressed relative to the F11 strain (a common strain in South Africa), in contrast to the typical H37Rv strain used in this work and for most structure determination, it was observed that some of the target residues of the mutations were already part of the models. In these cases, the reverse mutation was generated and used as ‘wild type’ instead.

#### Software

Mutations were analysed by the programs SDM and mCSM, which consider complementary features that predict the likely effect that a point mutation will have on a given structure in terms of stability (or in binding to other macromolecules, in the case of mCSM). The stability of a protein is typically quantified in terms of the free energy of unfolding, so the predicted changes in stability are expressed as estimates of the ΔΔ*G* between the native and the mutant forms of the proteins.

SDM is based on a statistical potential energy function developed by Topham et al. (12), which uses environment-specific substitution frequencies in families of homologous proteins to determine a stability score, from which a ΔΔ*G* value can be derived. SDM requires a model of the mutant protein for its operation, so the program Andante (38) was used to perform the mutation *in silico*. Andante uses environment-specific libraries of rotamers in conjunction with optimisation algorithms to address the issue of side-chain placement in comparative models, while limiting the problem of the explosive combinatorial complexity of rotamer conformations. In this case, it was used for its mutation function, which is designed for the replacement of individual residues.

mCSM (15) extends the concepts used on Pires et al. (39) to generate a signature encoding the interatomic distance patterns and local environment of a particular mutation in a structure. Based on a machine-learning model built from the application of this signature on a training dataset, a ΔΔ*G* estimate can be generated.

### Web interface and API

A very important factor for the usefulness of a database is the accessibility and user-friendliness of its interface. A clean and versatile web interface and RESTful API for CHOPIN was developed according to modern web standards and is available at http://structure.bioc.cam.ac.uk/chopin/.

The main results can be accessed through its ‘Browse’ section, with all processed sequences colour-coded from red to cyan according to the confidence of their best FUGUE predictions (red for *Z*-scores <3.5; orange <4.0; yellow <6.0; green <15.0 and cyan ≥15.0). The list can be filtered on the fly according to keywords. The page for each gene includes basic information about the sequence along with links for more detailed annotation from TB Database (http://www.tbdb.org; 16), TubercuList (http://tuberculist.epfl.ch; 40) and WebTB (http://www.webtb.org) and UniProt (http://www.uniprot.org; 41). Any FUGUE hits for the protein are displayed with their significance and the covered range of the sequence. Additionally, if there exist any experimental structures for the protein, they are displayed and linked to, along with some essential information such as number of chains in the PDB, experimental method, crystallographic resolution (if available) and range of coverage.

Each hit has a page with an overview of the different alignments and models that were generated according to the various conformational states of any available templates. This includes information such as the length, coverage and PID of the alignment, template names and estimated quality of the models. The alignments can viewed directly in JOY (42) format or downloaded in both JOY and PIR, but also have their own detailed page where models can be individually downloaded or viewed inline in 3D, coloured according to the predicted quality of each region, as estimated by DOPE, and further information about the template and the models is displayed, such as details of the quality assessment.

In addition to a persistent search field that allows finding a sequence quickly according to various known identifiers, the interface also provides an advanced search form to display a filtered list of sequences and results according to various criteria such as FUGUE scores, model quality, PID, coverage, length, homologous families and conformational state.

Another feature of CHOPIN is a comprehensive and automatically updated registry of all published experimental structures of *Mtb* with their associated gene names, basic information about the structure and function and direct download links. Annotation for ligands and structural interactions are also available via CREDO.

The results of the mutation analysis are available in their own section, where they can be filtered according to any criteria or the models viewed with the mutated residue conveniently highlighted for illustration.

Finally, models and their metadata are available for direct or programmatic access using RESTful URLs. In its simplest form, the best model in terms of coverage and quality and the complete metadata for a given sequence are made available to the user, giving priority to any available experimental structures. Additionally, it is possible to specify a particular residue that should be covered by the model or a specific template conformational state, to obtain a different model. Metadata for the models is provided in JSON format. The details about the API implementation are available at the website.

## Results and discussion

**Figure 2.**
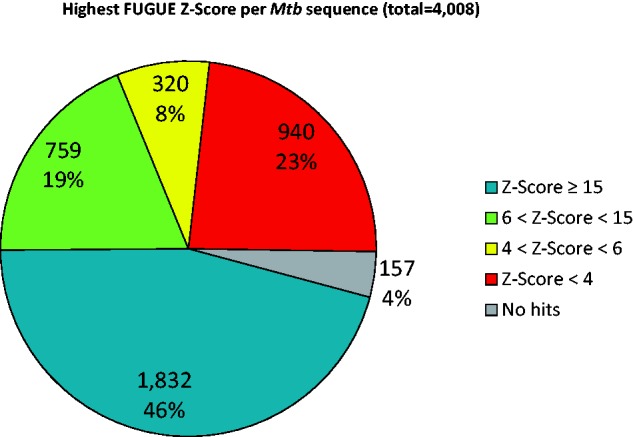
Distribution of the best FUGUE *Z*-Scores for all sequences of Mtb proteome. Blue (*Z*-Score ≥ 15), green (6 < *Z*-Score < 15) and yellow (4 < *Z*-Score < 6) correspond to very high, high and reasonable confidence matches, respectively, whereas red indicates non-significant hits.

As displayed on Fig. 2, FUGUE was able to find significant hits for 2911 of the 4008 sequences in the proteome, corresponding to roughly 73%. Of those, 759 (19%) had high confidence hits (FUGUE *Z*-Score ≥6, <15) and 1832 (46%) very high confidence (FUGUE *Z*-Score ≥15) ones. No reliable hits were found for 1097 of the sequences.

**Figure 3.**
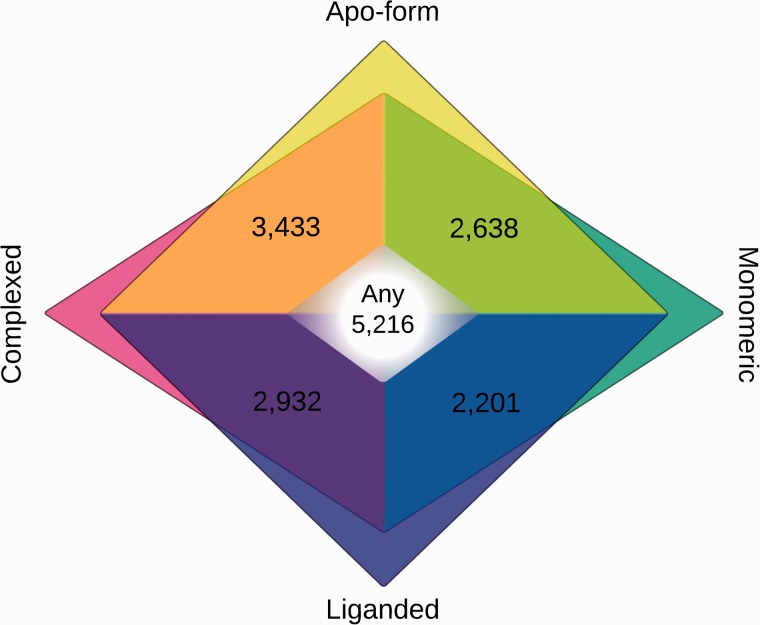
Venn diagram of number of alignments according to conformational state of templates. Alignments in apo-form are in yellow tones; liganded in blue tones; monomeric in teal and complexed in magenta. State-free alignments, where templates can be in any state, are shown in the white centre.

Table 1 details the statistics from the modelling results further. There were 5268 significant hits across all proteins, as many of them had more than one non-redundant hit (i.e. covering a different region of the sequence), indicating the existence of multiple domains. The distribution of conformational states is illustrated on Fig. 3. About 10% of these hits, 523, corresponded to multi-domain profiles from TOCCATA, suggesting that the proteome contains combinations of domains either not yet published or classified, or not detected by FUGUE.

**Table 1.** General statistics of CHOPIN pipeline results

Category	Count
Sequences w/ FUGUE *Z*-Score ≥15	1832
Sequences w/ FUGUE *Z*-Score ≥6, <15	759
Sequences w/ FUGUE *Z*-Score ≥4, <6	157
Sequences w/ FUGUE *Z*-Score <4	759
Sequences without FUGUE hits	157
Number of significant hits (*Z*-Score ≥4)	5268
Unique TOCCATA profiles among hits	2009
Number of multi-domain hits	523
Number of alignments	16 420
Number of unique alignments	13 169
Alignments w/apo-form templates	6071
Alignments w/liganded templates	5133
Alignments w/complexed templates	6365
Alignments w/monomeric templates	4839
Alignments w/templates in any state	5216
Average template PID (%)	24.21
Total number of models	49 218
Top models w/ ‘great’ quality rating (=4)	7026
Top models w/ ‘good’ quality rating (≥3, <4)	3187
Top models w/ ‘fair’ quality rating (≥2, <3)	2269
Top models w/ ‘poor’ quality rating (<2)	3931

A total of 16 420 alignments was constructed, although only 13 169 were unique, since in some cases the state-free alignment would be the same as one of the others. In 43% (7026) and 19% (3187) of all alignments, the best models generated were assigned a ‘great’ or ‘good’ quality rating, respectively, and 14% (3187) and 24% (2269) falling under the ‘fair’ and ‘poor’ categories. When considering only the best model per hit (i.e. independently of state), the percentages are 53, 19, 13 and 15% for ‘great’, ‘good’, ‘fair’ and ‘poor’ models, respectively, suggesting that the choice of template and alignment can make a significant difference in model quality. On a per sequence basis, the percentages are 60, 16, 12 and 12%.

Table 2 shows the 44 mutations predicted by either SDM or mCSM to be ‘deleterious’ (defined as having an absolute ΔΔ*G* value >2 kJ/mol) on models of at least ‘fair’ quality. Of those, 11 correspond to mutations that are either of the high confidence TBDReaMDB set or only on the MDR or XDR ones, while the rest correspond to mutations present in all strains. The full list of mutations and the analysis results is on Supplementary Table 2 and on the website.

**Table 2.** Mutations predicted to be deleterious to protein stability according to SDM and mCSM

Sequence ID	Mutation	Strain/Source	Sequence Description	SDM ΔΔG (kJ/mol)	mCSM ΔΔG (kJ/mol)
Rv0006	A74S	FLQ	DNA gyrase subunit A gyrA	−2.29	−1.15
Rv0006	D94A	FLQ	DNA gyrase subunit A gyrA	2.04	−0.79
Rv0006	G247S	DS,MDR,XDR	DNA gyrase subunit A gyrA	−3.28	−1.29
Rv0237	A240V	DS,MDR,XDR	Lipoprotein lpqI	2.18	−0.71
Rv0319	G69D	DS,MDR,XDR	Pyrrolidone-carboxylate peptidase pcp	−1.57	−2.31
Rv0404	P478H	DS,MDR,XDR	Fatty-acid-CoA ligase fadD30	1.38	−2.10
Rv0655	V144A	DS,MDR,XDR	Ribonucleotide transport ATP-binding protein ABC transporter mkl	−1.53	−2.38
Rv0667	L456S	DS,MDR,XDR	DNA-directed RNA polymerase beta subunit rpoB	−4.11	−2.66
Rv0667	I1112T	XDR	DNA-directed RNA polymerase beta subunit rpoB	−4.53	−2.43
Rv0721	A105V	DS,MDR,XDR	30. ribosomal protein S5 rpsE	2.18	−0.25
Rv0790c	F83S	DS,MDR,XDR	Hypothetical protein	−2.20	−2.66
Rv1001	T281M	DS,MDR,XDR	Arginine deiminase arcA	2.39	−0.31
Rv1039c	A67T	DS,MDR,XDR	PPE family protein	−2.48	−0.92
Rv1240	G306R	DS,MDR,XDR	Malate dehydrogenase mdh	3.41	−0.97
Rv1276c	Q79E	DS,MDR,XDR	Hypothetical protein	−0.31	−2.48
Rv1569	A171G	DS,MDR,XDR	8.Amino-7-oxononanoate synthase bioF1	−2.24	−1.39
Rv1600	S271A	DS,MDR,XDR	Histidinol-phosphate aminotransferase hisC1	2.85	−0.50
Rv1605	G145V	DS,MDR,XDR	Cyclase hisF	2.55	−0.41
Rv1638	S908I	DS,MDR,XDR	Excinuclease ABC subunit A (DNA-binding ATPase) uvrA	3.02	0.11
Rv1825	P181S	DS,MDR,XDR	Hypothetical protein	−0.81	−2.03
Rv1870c	D123G	DS,MDR,XDR	Hypothetical protein	2.51	−0.38
Rv1878	S296F	DS,MDR,XDR	Glutamine synthetase glnA3	3.03	−0.90
Rv1933c	V196A	MDR,XDR	Acyl-CoA dehydrogenase fadE18	−2.73	−2.53
Rv2000	L275P	XDR	Hypothetical protein	−6.18	−0.95
Rv2043c	A3P	PZA	Pyrazinamidase/Nicotinamidase PncA (PZase)	−3.35	−0.51
Rv2043c	Q10P	PZA	Pyrazinamidase/Nicotinamidase PncA (PZase)	−2.32	−0.49
Rv2043c	C14H	PZA	Pyrazinamidase/Nicotinamidase PncA (PZase)	−4.49	−1.44
Rv2043c	C14R	PZA	Pyrazinamidase/Nicotinamidase PncA (PZase)	−3.76	−0.63
Rv2043c	L19P	PZA	Pyrazinamidase/Nicotinamidase PncA (PZase)	−2.48	−1.46
Rv2043c	V21G	PZA	Pyrazinamidase/Nicotinamidase PncA (PZase)	−4.20	−1.60
Rv2043c	Y34S	PZA	Pyrazinamidase/Nicotinamidase PncA (PZase)	−2.47	−2.96
Rv2122c	A88D	DS,MDR,XDR	Phosphoribosyl-ATP pyrophosphohydrolase hisE	−2.70	−0.82
Rv2161c	G105A	DS,MDR,XDR	Hypothetical protein	2.23	−0.47
Rv2197c	P112S	DS,MDR,XDR	Conserved transmembrane protein	2.77	−0.56
Rv2250c	A119T	DS,MDR,XDR	Hypothetical transcriptional regulatory protein	−2.02	−0.68
Rv2464c	A99T	DS,MDR,XDR	Hypothetical DNA glycosylase	−2.84	−1.35
Rv2886c	V153A	DS,MDR,XDR	Hypothetical resolvase	−2.73	−2.48
Rv2887	S2G	DS,MDR,XDR	Hypothetical transcriptional regulatory protein	2.58	−0.24
Rv3032	Q310L	DS,MDR,XDR	Hypothetical transferase	3.07	−0.33
Rv3174	L42R	DS,MDR,XDR	Hypothetical short-chain type dehydrogenase/reductase	−2.32	−1.56
Rv3545c	I359T	DS,MDR,XDR	Cytochrome P450 125 cyp125	−2.20	−2.79
Rv3591c	F30S	DS,MDR,XDR	Hypothetical hydrolase	−3.05	−1.96
Rv3606c	L172P	DS,MDR,XDR	2.Amino-4-hydroxy-6- hydroxymethyldihydropteridine pyrophosphokinase folk	−2.74	−1.45
Rv3719	R310T	DS,MDR,XDR	Hypothetical protein	−2.20	−1.80

DS (Drug Sensitive), MDR (Multiple Drug Resistant) and XDR (eXtensively Drug Resistance) refer to the KwaZulu-Natal strains sequenced by the Broad Institute, with residue numbers given relative to the F11 reference strain. PZA and FLQ indicate to various high-confidence pyrazinamide or fluoroquinone resistant strains, respectively, as identified on TBDreaMDB, with residue numbers relative to the H37Rv strain

However, there are various mechanisms of resistance to a drug: of these SDM and mCSM estimate the effect of a mutation on the structural stability of the protein, which in turn may affect drug binding. Mutations that generate resistance by directly interfering with the binding of a drug molecule can detected noting their location with respect to the drug-binding site; quantitative methods trained using the database Platinum (Pires D, Ascher D and Blundell TL, under review) and graph signatures are under development and will be incorporated later.

An example of resistance-conferring mutations that act through disrupting the stability of a protein can be found in Rv2043c/*pncA*. This gene encodes for the nicotinamidase/ pyrazinamidase responsible for the conversion of the pro-drug pyrazinamide into its active form pyrazinoic acid, such that disrupting either the function or stability of the enzyme would lead to preventing the drug from becoming active. Indeed, various mutations across the gene, including deletions, truncations and frame shifts, have been shown to confer resistance to pyrazinamide (43). In particular, Petrella et al. (44) have shown structurally how a number of mutations that lead to a loss of stability affect the catalytic activity of the enzyme. None of the nine mutations on the TBDReaMDB high-confidence PZA set are part of the active site as proposed on their paper, but seven out of them were predicted to be deleterious by at least one of the programs.

## Conclusion and future perspectives

The CHOPIN database provides a resource for structural information on Mtb, including a flexible and user-friendly repository of high quality homology models and domain annotations, as well as up-to-date experimental structures. Its homology recognition step has helped in enriching the functional annotation of the proteome (45) and its models assisted in elucidating the mechanism of action of potential drugs (46). Its focus on providing a variety of models based on specific conformational states of the templates is, as far as we know, unique and should prove valuable to applied researchers in the field, despite the necessary simplifications that were adopted to deal with the highly complex topic of conformational variability. We aim to perform updates following those of the underlying profile and template database, TOCCATA, which itself relies on the SCOP and CATH release schedule of every year or so. We intend to hone our methods to provide more refined and flexible results, such as fully modelled complexes and specific ligands.

The structural analysis of polymorphisms, while currently limited in scope, should also be of interest to researchers in drug discovery. Our group is currently working on further methods to expand and improve the predictions of the effect of structural changes, and as better databases of polymorphisms become available (47, 48), we aim to expand our database with their analysis.

## Supplementary Material

Supplementary Data
