# Supplementary material for: CHOPIN: a web resource for the structural and functional proteome of Mycobacterium tuberculosis
Source: Database (Oxford). 2015 Mar 31;2015:bav026. doi: 10.1093/database/bav026 (PMC4381106; doi:10.1093/database/bav026)
Supplement: Supplementary Data [file supp_2015_bav026_index.html]

CHOPIN: a web resource for the structural and functional proteome of Mycobacterium tuberculosis — Supplementary Data 

# CHOPIN: a web resource for the structural and functional proteome of *Mycobacterium tuberculosis*

## Supplementary Data

files

**Files in this Data Supplement:**

- Supplementary Data - xlsx file
- Supplementary Data - docx file
