# Supplementary material for: CHOPIN: a web resource for the structural and functional proteome of Mycobacterium tuberculosis
Source: Database (Oxford). 2015 Mar 31;2015:bav026. doi: 10.1093/database/bav026 (PMC4381106; doi:10.1093/database/bav026)
Supplement: Supplementary Data [file supp_bav026_New_Microsoft_Office_Word_Document.docx]

**Supplementary Table S1:** Detailed threshold values from quality methods for fine-grained scoring of model quality.

**Supplementary Table S2:** Full list of mutations considered for analysis. DS (Drug Sensitive), MDR (Multiple Drug Resistant) and XDR (eXtensively Drug Resistance) refer to the KwaZulu-Natal strains sequenced by the Broad Institute, with residue numbers given relative to the F11 reference strain. PZA and FLQ indicate to various high-confidence pyrazinamide or fluoroquinone resistant strains, respectively, as identified on TBDreaMDB, with residue numbers relative to the H37Rv strain.
